# Hybrid Deployment Optimization Algorithm for Reconfigurable Intelligent Surface

**DOI:** 10.3390/s25237195

**Published:** 2025-11-25

**Authors:** Yifan Lin, Xinwei Lin, Zhiyu Han, Yafeng Wang

**Affiliations:** 1 The Key Laboratory of Universal Wireless Communications, Ministry of Education, Beijing University of Posts and Telecommunications, Beijing 100876, China; yflin@bupt.edu.cn (Y.L.); hanzhiyu@bupt.edu.cn (Z.H.); 2 The Future Research Laboratory, China Mobile Research Institute, Beijing 100053, China; linxinwei@chinamobile.com

**Keywords:** reconfigurable intelligent surface, deployment optimization, two-stage branch-and-bound method, coverage blind spots, hybrid algorithm

## Abstract

As a key 6G candidate technology, reconfigurable intelligent surface (RIS) integrates into sensor-communication systems, supporting positioning and sensing as environmental sensor nodes or anchors. To address efficient RIS deployment under constraints and mitigate wireless communication blind spots, this paper proposes a hybrid optimization algorithm. It decomposes the NP-hard combinatorial optimization problem into two stages: (1) a greedy strategy ensures coverage completeness by allocating one locally optimal RIS to each independent shadow area; (2) a Branch-and-Bound (BnB) algorithm optimizes global deployment to maximize overall signal gain in shadow areas. This decoupling reduces computational complexity for large-scale problems. Simulation results show the algorithm’s superiority: the greedy phase guarantees fair coverage, and the BnB-based global optimization achieves up to 56.85% higher average Signal-to-Interference-plus-Noise Ratio (SINR) gain in shadow areas than random deployment, improving both shadow-area user communication quality and overall network performance.

## 1. Introduction

In dense urban environments, severe signal attenuation caused by high-rise buildings leads to extensive coverage blind spots [1], known as “shadow areas”, significantly degrading communication quality for non-line-of-sight (NLoS) users [2]. To address this challenge, RIS technology [3]—as a novel wireless environment control technique—has drawn widespread attention from academia and industry in recent years [4].

To maximize the performance gains offered by RIS, optimizing their deployment locations is crucial. Extensive research has been conducted on RIS deployment optimization. Existing studies broadly fall into three categories: some employ heuristic algorithms, such as Deb and Ghosh’s use of a greedy strategy to overcome static obstacles in millimeter-wave direct communication [5], and Lu et al.’s combination of grid partitioning with heuristic optimization to enhance deployment efficiency [6], while Zhang et al. proposed an adaptive neighborhood search RIS deployment method incorporating particle swarm optimization [7] to find suboptimal solutions within reasonable time frames. However, such methods cannot guarantee optimality. Another category relaxes discrete position variables into continuous ones and applies convex optimization techniques like alternating optimization, such as the joint active-passive beamforming alternating optimization method [8]. In single-base-station, single-user RIS-assisted wireless communication networks [9], coverage analysis is performed by optimizing the direction and horizontal distance of reflectors. Yu et al. explored convex optimization modeling for RIS-assisted MISO scenarios [10]. Recently, Xie et al. further characterized the relationship between RIS coverage and the number of units [11], while Wang et al. investigated deployment optimization in ultra-large-scale RIS systems [12]. These approaches enhance explainability but often fall into local optima, and variable relaxation may cause performance degradation. Recent research has begun exploring deep learning (DL)-based approaches [13,14], where deep learning or neural networks learn the mapping relationship between environmental features and optimal deployment locations. In the more complex STAR-RIS scenario, Gao et al. proposed using deep reinforcement learning to achieve joint optimization of coverage and capacity [15]. While these methods can capture complex environmental features, they generally require significant training overhead and exhibit limited generalization capabilities in dynamic environments.

It is noticed that the aforementioned heuristic, convex optimization, and deep learning iterative optimization methods predominantly treat RIS deployment as a complex numerical optimization problem, yet overlook a fourth critical approach: the closed-form solution deployment approach. Such methods do not rely on iterative suboptimal solutions, instead simplifying the model through channel customisation: deploying RIS along the discrete Fourier transform (DFT) directions of the base station (BS), actively reshaping the wireless channel into a ‘deterministic’ structure characterised by orthogonality and sparsity [16,17]. This strategy significantly simplifies the joint Tx-RIS-Rx design, enabling the customised channel to bypass complex matrix decomposition and directly derive low-complexity precoding and phase shifter closed-form solutions [16]. Furthermore, it substantially reduces CSI overhead, requiring only feedback of RIS directional parameters (e.g., AoD/AoA) rather than the full channel matrix.

However, its limitations also stem from reliance on specific conditions: on one hand, deployment flexibility is constrained, as the RIS must precisely align with the DFT direction, and building obstructions in practical scenarios can compromise positional accuracy [18]. Secondly, it exhibits high sensitivity to channel conditions, as the closed-form solution relies on line-of-sight (LoS) paths being dominant. If NLoS scattering intensifies, the degradation of channel orthogonality leads to performance deterioration, necessitating path pruning to select strong paths and maintain effectiveness [16]. Evidently, such methods sacrifice partial deployment universality in exchange for simplified system design and low complexity.

Despite significant progress in performance optimization, a common limitation is that most studies focus solely on single global metrics like aggregate network rate or spectral efficiency [19], neglecting practical network planning constraints such as quality of service (QoS), particularly coverage fairness. Addressing this, Huang et al. proposed beamforming design based on minimax fairness in STAR-RIS scenarios to enhance edge user experience [20]. However, without coverage constraints, optimization results may lead to excessive signal strength in some areas while failing to effectively improve critical shadow areas. Therefore, solving this NP-hard problem becomes more challenging under the stringent constraint that all shadow areas must be covered.

To address these limitations, this paper proposes an innovative hybrid optimization algorithm framework specifically designed for RIS deployment optimization under coverage constraints. Specifically, this complex combinatorial optimization problem is decomposed into two core subproblems: ensuring coverage completeness for all shadowed areas and, on this basis, improving overall network performance. These subproblems are solved sequentially through a staged strategy. The main contributions of this paper are summarized as follows:

- A novel hybrid optimization algorithm framework is proposed. This framework integrates a two-stage optimization approach combining a greedy heuristic strategy with a branch-and-bound algorithm. It achieves overall network performance optimization while ensuring effective coverage of all shadow areas, significantly reducing computational complexity.
- The first stage employs a local optimization method based on a greedy strategy to assign locally optimal RIS positions for each independent shadow area, strictly satisfying coverage completeness constraints with low computational overhead.
- The second stage introduces an efficient branch-and-bound algorithm for global optimization of remaining RIS resources after basic coverage is achieved. Combining marginal gain evaluation with pruning mechanisms, this algorithm efficiently explores the vast search space to obtain RIS deployment schemes approaching global optimality.
- Simulation results demonstrate that the proposed method achieves significantly higher average SINR in shadowed areas compared to random deployment strategies, fully validating its effectiveness in enhancing network coverage performance in complex real-world environments.

## 2. System Model

This paper considers an RIS-assisted multi-cell downlink cellular communication system comprising a central base station and its six adjacent base stations, denoted collectively as B. Randomly distributed user equipment (UE) exists in the system, whose set is denoted as U. To enhance signal coverage performance in specific areas, a set C of predefined RIS candidate deployment locations is established in the environment. Due to obstructions such as urban buildings, some users lack direct LoS links with the base stations, forming NLOS shadow areas.

### 2.1. Channel Model

Assume the service base station is equipped with M antennas and uses beamforming vector $w\in C^{M\times 1}$ to transmit symbol *s* to a single-antenna user. The total signal received by the user is the superposition of the direct path signal and the RIS-assisted cascaded reflected path signal, which can be expressed as [21]:

(1) $$y=(h_{d}^{H}+h_{ru}^{H}\Phi h_{br})ws+n$$

(2) $$h_{d}=\sum_{l=0}^{L-1}\alpha_{l}a_{\mathrm{BS}}\left(\theta_{l}\right)$$

(3) $$h_{ru}=\sum_{m=0}^{M-1}\beta_{m}a_{\mathrm{RIS}}\left(\phi_{m}\right)$$

(4) $$h_{br}=\alpha_{\mathrm{LOS}}a_{\mathrm{RIS}}\left(\phi_{\mathrm{LOS}}\right)a_{\mathrm{BS}}^{H}\left(\theta_{\mathrm{LOS}}\right)+\sum_{l=1}^{L^{\prime }-1}\alpha_{l}a_{\mathrm{RIS}}\left(\phi_{l}\right)a_{\mathrm{BS}}^{H}\left(\theta_{l}\right)$$

Among these, $h_{d}\in C^{M\times 1}$ is the direct channel vector from the BS to the UE, $h_{br}\in C^{N\times M}$ is the channel matrix from the BS to the RIS with N reflectors, $h_{ru}\in C^{N\times 1}$ is the channel vector from the RIS to the UE. In (2), (3), and (4), $L,L^{\prime },M$ denote the number of multipaths for the corresponding paths, while $\alpha_{l},\beta_{m},\alpha_{LOS}$ represent the complex gain corresponding to the path respectively. $a_{\mathrm{RIS}}$ and $a_{\mathrm{BS}}$ denote the array response vectors. All parameters conform to [22]. $\Phi \in C^{N\times N}$ is the phase shift response matrix of the RIS, and $n∼\mathrm{CN}\left(0,\sigma^{2}\right)$ represents additive white Gaussian noise (AWGN). Therefore, user u receives the signal from base station k as shown in (5), where $h^{H}=h_{d}^{H}+h_{ru}^{H}\Phi h_{br}$. This enables users to calculate the SINR using (6) to assess the quality of the received signal, where $P=E[|s{|}^{2}]$.

(5) $$y_{k,u}=h_{k,u}^{H}w_{k,u}s_{k,u}+\sum_{\begin{array}{c} j=1\\ j\neq k \end{array}}^{K}h_{j,u}^{H}w_{j,u}s_{j,u}+\sum_{\begin{array}{c} p=1\\ p\neq u \end{array}}^{Q}h_{k,u}^{H}w_{k,p}s_{k,p}+n_{k,u}$$

(6) $${\mathrm{SINR}}_{k,u}=\frac{|h_{k,u}^{H}w_{k,u}{|}^{2}P_{k,u}}{\sum_{\begin{array}{c} j=1\\ j\neq k \end{array}}^{K}|h_{j,u}^{H}w_{j,u}{|}^{2}P_{j,u}+\sum_{\begin{array}{c} p=1\\ p\neq u \end{array}}^{Q}{\left|h_{k,u}^{H}w_{k,p}\right|}^{2}P_{k,p}+\sigma^{2}}$$

The core functionality of RIS lies in its ability to modulate incident signals through its phase-shifting matrix Φ [23]. Φ is a diagonal matrix of N×N size, expressed as Equation (7), where $\theta_{n}\in \left[0,2\pi \right)$ is the controllable phase shift introduced by the n-th unit, and $\beta_{n}\in \left[0,1\right]$ is the corresponding reflection amplitude. Under the assumption of an ideal passive RIS, the signal is perfectly reflected, and the amplitude of all reflection units is $\beta_{n}=1,\forall n\in \left\{1,\ldots ,N\right\}$. Therefore, the phase matrix of the ideal RIS can be simplified to the rightmost term of Equation (7).

(7) $$\Phi =\mathrm{diag}\left(\beta_{1}e^{j\theta_{1}},\beta_{2}e^{j\theta_{2}},\ldots ,\beta_{N}e^{j\theta_{N}}\right)=\mathrm{diag}\left(e^{j\theta_{1}},e^{j\theta_{2}}\ldots ,e^{j\theta_{N}}\right)$$

By jointly optimizing the deployment locations of RISs and their phase shifts $\left\{\theta_{n}\right\}$, the system can construct high-quality virtual LoS links under NLoS conditions, thereby effectively enhancing signal strength in the target area.

### 2.2. Shadow Area Modeling

**Figure 1:**
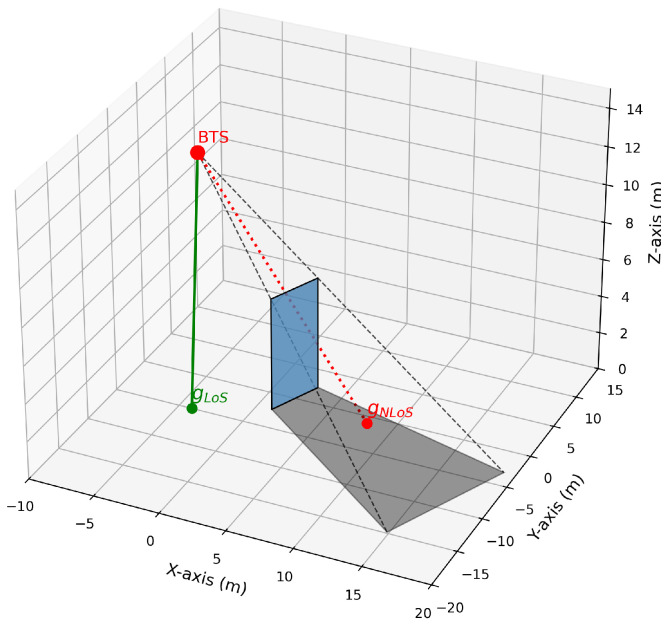
The Process of Shadow Generation.

The specific characteristics of the channel vector (such as $h_{d}$) depend on the propagation conditions of the link. This paper employs a geometric approach to model shadow areas: obstacles are abstracted as planar entities with specific height and width. Each base station antenna is treated as a point radiation source, with obstacles casting shadow areas onto the ground (z=0 plane). As shown in Figure 1, the boundary of the shadow area is defined by the base station location, the geometric vertices of the obstacle, and its projection onto the ground.

Let the union of all shadow areas be denoted as $S=\left\{s_{1},s_{2},\ldots ,s_{K}\right\}$. For any user u∈U, its location is denoted as $p_{u}$. If $p_{u}$ is located within a shadow area $s_{k}\in S$ associated with base station b∈B, the direct link between BS and UE is classified as an NLoS link; otherwise, it is classified as an LoS link. Channel vectors corresponding to different link states follow distinct path loss and fading distributions. This characteristic directly influences the specific values of channel vectors and serves as the core basis for RIS deployment optimization.

## 3. Algorithm

Based on the established system model, this section focuses on solving an RIS deployment optimization problem with coverage constraints. The objective is to ensure effective coverage of all shadowed areas under limited RIS resources while maximizing overall system performance. Due to the discrete nature of candidate locations and the complexity of coverage constraints, this problem inherently belongs to the class of NP-hard combinatorial optimization problems, making it difficult to solve via direct enumeration or traditional convex optimization methods. To address this, this paper proposes a phased hybrid optimization algorithm. By decomposing the problem and implementing a two-stage joint solution approach, it achieves efficient near-optimal solutions for the coverage-constrained RIS deployment problem.

### 3.1. Problem Modeling

Let the set of RIS candidate locations be denoted as $C=\{c_{1},c_{2},\ldots ,c_{N}\}$. Define the binary deployment decision vector $x={\left[\begin{array}{c} x_{1},x_{2},\ldots ,x_{N} \end{array}\right]}^{T}$ such that $x_{i}=1$ indicates deployment of RIS at candidate location $c_{i}$, while $x_{i}=0$ indicates no deploying. For any user *u* in the shadow area, its received power depends on the deployment scheme x. When no RIS is deployed (x=0), received power of user *u* equals its baseline direct-path power $P_{u}\left(0\right)$. Under deployment scheme x, its total received power $P_{u}\left(x\right)$ can be calculated based Equation (1). Thus, the signal gain brought by deployment scheme x to user *u* is $\Delta P_{u}\left(x\right)=P_{u}\left(x\right)-P_{u}\left(0\right)$.

Therefore, the RIS deployment optimization problem can be formalized as Equation (8):

(8) $$\begin{array}{c} \max_{x}G\left(x\right)=\sum_{u\in U_{\mathrm{shadow}}}\Delta P_{u}\left(x\right)\\ s.t.\sum_{i=1}^{N}x_{i}\leq R_{\max}\\ \sum_{i\in C_{j}}x_{i}\geq 1,\forall s_{j}\in S\\ x_{i}\in \left\{0,1\right\},\;\forall c_{i}\in C \end{array}$$

Among these, G(x) represents the total gain for all shadow area users, $U_{\mathrm{shadow}}$ denotes the set of all shadow area users, $R_{\max}$ indicates the maximum number of RISs permitted for deployment, and $C_{i}$ signifies the subset of candidate RISs considered capable of serving the shadow area $s_{j}$. Each shadow area is covered by at least one RIS, meaning the coverage constraint is strictly satisfied.

### 3.2. Hybrid Optimization Algorithm Framework

To address the aforementioned optimization problem, this paper proposes a two-stage hybrid optimization algorithm, whose detailed procedures are outlined in Algorithms 1 and 2. The method first satisfies coverage constraints through a greedy strategy, followed by global optimization using branch-and-bound.
**Algorithm 1** Hybrid RIS Deployment Optimization Algorithm—Main Procedure**Require:** Set of RIS candidate locations C; Set of shadow areas S; Maximum number of deployments $R_{\max}$**Ensure:** The final RIS deployment scheme $X_{\mathrm{final}}$ 1:**procedure** HybridDeploy($C,S,R_{\max}$)                   ▹**Phase 1: Greedy selection to satisfy coverage constraints** 2:       $X_{\mathrm{initial}}\leftarrow \emptyset$ 3:       **for** each shadow area $s_{j}\in S$ **do** 4:        $C_{j}\leftarrow \mathrm{FindCandidatesFor}\left(s_{j}\right)$                  ▹ Find candidate RISs for $s_{j}$ 5:        $c_{j}^{*}\leftarrow \arg\max_{c_{i}\in C_{j}}G\left(c_{i}|s_{j}\right)$             ▹ Select the RIS with max local gain 6:        $X_{\mathrm{initial}}\leftarrow X_{\mathrm{initial}}\cup \left\{c_{j}^{*}\right\}$ 7:       **end for**                  ▹**Phase 2: Branch and Bound for global gain maximization** 8:       $R_{\mathrm{rem}}\leftarrow R_{\max}-\left|X_{\mathrm{initial}}\right|$                     ▹ Calculate remaining slots 9:       $C_{\mathrm{rem}}\leftarrow C\setminus X_{\mathrm{initial}}$                      ▹ Get the remaining candidate set10:       **if** $R_{\mathrm{rem}}>0$ **then**11:        Sort $C_{\mathrm{rem}}$ in descending order by Marginal Gain12:        $G_{\mathrm{best}}\leftarrow -\infty$13:        $X_{\mathrm{best\_bnb}}\leftarrow \emptyset$14:        BnB-DFS($C_{\mathrm{rem}}$, $R_{\mathrm{rem}}$, *∅*, 0, $G_{\mathrm{best}}$, $X_{\mathrm{best\_bnb}}$)              ▹ Call Algorithm 215:        $X_{\mathrm{final}}\leftarrow X_{\mathrm{initial}}\cup X_{\mathrm{best\_bnb}}$16:       **else**17:        $X_{\mathrm{final}}\leftarrow X_{\mathrm{initial}}$18:       **end if**19:       **return** $X_{\mathrm{final}}$20:**end procedure**
**Algorithm 2** Auxiliary Procedure for Algorithm 1: Branch and Bound Search (BnB-DFS) 1:**procedure** BnB-DFS($C^{\prime },k,X_{\mathrm{curr}},\mathrm{idx},\mathrm{ref}\;G_{\mathrm{best}},\mathrm{ref}\;X_{\mathrm{best}}$) 2:      **if** $|X_{\mathrm{curr}}|=k$ **then**                       ▹ A complete solution is found 3:         current_gain $\leftarrow \mathrm{CalculateTotalGain}(X_{\mathrm{curr}})$ 4:         **if** current_gain $>G_{\mathrm{best}}$ **then** 5:            $G_{\mathrm{best}}\leftarrow$ current_gain 6:            $X_{\mathrm{best}}\leftarrow X_{\mathrm{curr}}$ 7:         **end if** 8:         **return** 9:      **end if**10:    **if** idx $\geq |C^{\prime }|$ **then**                    ▹ All candidates have been traversed11:        **return**12:    **end if**                                    ▹**Bounding and Pruning**13:    $k_{\mathrm{needed}}\leftarrow k-\left|X_{\mathrm{curr}}\right|$14:    $UB\leftarrow \mathrm{CalculateTotalGain}\left(X_{\mathrm{curr}}\right)+\sum_{i=\mathrm{idx}}^{\mathrm{idx}+k_{\mathrm{needed}}-1}\mathrm{MG}\left(C_{i}^{\prime }\right)$     ▹ Calculate Upper Bound15:    **if** $UB\leq G_{\mathrm{best}}$ **then**16:         **return**               ▹ Prune: this branch cannot produce a better solution17:    **end if**                                            ▹**Branching**18:   ▹ Branch 1: Do not select the current candidate $C_{\mathrm{idx}}^{\prime }$ BnB-DFS($C^{\prime },k,X_{\mathrm{curr}},\mathrm{idx}+1,G_{\mathrm{best}},X_{\mathrm{best}}$)19:               ▹ Branch 2: Select the current candidate $C_{\mathrm{idx}}^{\prime }$ (if slots are available)20:    **if** $|X_{\mathrm{curr}}|<k$ **then**21:       $X_{\mathrm{new}}\leftarrow X_{\mathrm{curr}}\cup \left\{C_{\mathrm{idx}}^{\prime }\right\}$ BnB-DFS($C^{\prime },k,X_{\mathrm{new}},\mathrm{idx}+1,G_{\mathrm{best}},X_{\mathrm{best}}$)22:    **end if**23:**end procedure**

#### 3.2.1. Phase 1: Coverage Constraint Satisfaction

In the first stage, the objective is to ensure that each shadow area $s_{j}\in S$ is covered by at least one RIS, thereby satisfying the coverage constraints. The specific approach is as follows: First, each candidate RIS $c_{i}\in C$ is divided according to the shadow area it primarily serves, constructing a local candidate set. Let $c_{i}$ be included in the candidate set $C_{j}$ for shadow area $s_{j}$. Subsequently, for each shadow area $s_{j}$, the local contribution of each RIS in its candidate set $C_{j}$ is evaluated. The local gain $g(c_{i}|s_{j})$ of candidate RIS $c_{i}$ to shadow area $s_{j}$ is defined as Equation (9):

(9) $$g\left(c_{i}|s_{j}\right)=\sum_{u\in U_{s_{j}}}\Delta P_{u}\left(x_{c_{i}}\right)$$

Among these, $U_{s_{j}}$ is the set of users within the shaded area $s_{j}$, and $x_{c_{i}}$ is a decision vector that deploys RIS only at the *i* position. Then, through greedy selection, each shaded area $s_{j}$ is assigned its locally optimal RIS $c_{j}^{*}$:

(10) $$c_{j}^{*}=\arg\max_{c_{i}\in C_{j}}g\left(c_{i}|s_{j}\right)$$

Aggregate all selected locally optimal RISs to form the initial deployment set $X_{\mathrm{initial}}=\left\{{c_{1}}^{*},{c_{2}}^{*}\ldots ,{c_{K}}^{*}\right\}$. This ensures that coverage constraints are strictly satisfied.

#### 3.2.2. Phase 2: Maximizing Overall Gains

After completing Phase 1, compute the number of remaining RISs to be deployed $R_{rem}=R_{max}-\left|X_{initial}\right|$ and the remaining candidate set $C_{rem}=C\setminus X_{initial}$. If $R_{rem}>0$, initiate the Branch-and-Bound algorithm to select the optimal $R_{\mathrm{rem}}$ RISs from $C_{\mathrm{rem}}$. To enhance computational efficiency and enable the algorithm to prioritize exploring more promising solution branches, first calculate the Marginal Gain for each candidate RIS $c_{i}$ in $C_{\mathrm{rem}}$. The Marginal Gain $\mathrm{MG}(c_{i})$ of $c_{i}$ is defined as Equation (11):

(11) $$\mathrm{MG}\left(c_{i}\right)=\sum_{u\in U_{\mathrm{shadow}}}\Delta P_{u}\left(x_{c_{i}}\right)$$

The fundamental concept of the BnB algorithm is to construct a decision binary tree and systematically traverse the solution space through depth-first search (DFS). At any node, the total gain of the current solution is denoted as $G(X_{initial}\cup X_{curr})$, and its upper bound (UB) can be estimated as Equation (12):

(12) $$UB\left(X_{\mathrm{curr}},C^{\prime }\right)=G\left(X_{\mathrm{initial}}\cup X_{\mathrm{curr}}\right)+\sum_{i=1}^{k}\mathrm{MG}\left(c_{i}^{\prime }\right)$$

Among these, $c_{i}^{\prime }$ denotes the *k* RISs with the highest marginal gains in $C^{\prime }$. Let $G_{\mathrm{best}}$ represent the gain of the best solution found during the search. At each node, apply the pruning rule:

(13) $$\mathrm{if}\;UB\left(X_{\mathrm{curr}},C^{\prime }\right)\leq G_{\mathrm{best}},\mathrm{then}\;\mathrm{prune}$$

If $UB\leq G_{\mathrm{best}}$, then the branch is pruned to avoid futile searches in subtree that does not contain the optimal solution. By recursively performing “branching” (selecting or not selecting the next candidate RIS) and “bounding” (pruning), the algorithm ultimately finds the globally optimal residual deployment scheme $X_{\mathrm{best}\text{\_}\mathrm{bnb}}$ within acceptable time. The final deployment scheme is merged from the results of two phases:

(14) $$X_{\mathrm{final}}=X_{\mathrm{initial}}\cup X_{\mathrm{best}\text{\_}\mathrm{bnb}}$$

The detailed process of this hybrid algorithm is described by the pseudocode of Algorithms 1 and 2.

### 3.3. Complexity and Convergence Analysis

To assess the computational feasibility of the proposed algorithm, this section conducts a detailed analysis of the computational complexity of the RIS deployment scheme. We grid the simulation scenario and define the system parameters: *M* denotes the total number of grid cells in the simulation scenario, *N* represents the total number of candidate RIS site locations, *K* indicates the target number of deployed RIS, and *S* signifies the number of shadowed areas. Random deployment serves as the baseline for performance comparison, exhibiting a time complexity of O(M·K). This complexity primarily stems from the evaluation process of computing performance across the *K* deployed RIS. In contrast, the BnB algorithm, designed to search for a global optimum, requires at worst traversing NK combinations. Furthermore, evaluating each leaf node demands O(M·N) time, resulting in a total search time complexity of O(NK·M·N). This proves unacceptable for medium-sized values of *N* and *K*. To address this exponential explosion, the proposed hybrid BnB algorithm with shadow constraints leverages environmental prior information. Through an O(N·M) greedy pre-selection phase, it locks in *S* locally optimal RIS solutions. successfully reducing the exponential term in the subsequent BnB search space from *N* to N−S. This significantly lowers the worst-case search time complexity to O(N−SK−S·M·(N−S)). Compared to the original problem (8) as a binary combinatorial optimisation problem with covering constraints (computational complexity $O(2^{N})$), the proposed algorithm exhibits a total complexity significantly lower than the exponential complexity of the original problem. This enables efficient solution in large-scale scenarios, transforming the original problem’s exponentially unsolvable complexity into an engineering-solvable polynomial- level complexity.

Random deployment, as a non-iterative method, lacks a convergence process. The quality of its solutions is entirely dependent on random probability, offering no guarantee of excellence. BnB theoretically guarantees convergence to the global optimum by traversing the entire solution space; however, its convergence speed is constrained by the vast state space NK. In practical large-scale scenarios, it often faces combinatorial explosion, preventing convergence within an effective time. In contrast, constrained hybrid branch-and-bound employs a strategy of initial greedy fixation followed by precise search. Although this sacrifices theoretical global optimality by forcibly fixing partial solutions, it successfully achieves rapid convergence to high-quality suboptimal solutions within an engineering-acceptable time by reducing the search space exponentially to N−SK−S. This approach reaches an optimal balance between performance and efficiency.

## 4. Simulation Results

This section validates the hybrid optimization algorithm proposed in Section 3 through simulation experiments to evaluate its performance in RIS-assisted cellular networks. The experiments first describe the simulation environment setup, followed by a comparative analysis of the proposed algorithm, random deployment scheme, and RIS-free baseline scheme across key performance metrics, thereby comprehensively demonstrating the advantages of the proposed method.

**Figure 2:**
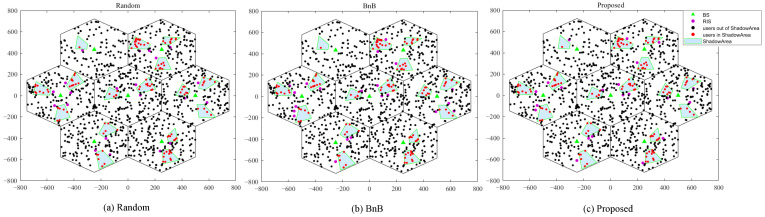
System Layout Diagram.

To construct a realistic urban macrocell communication scenario, the simulation employs a classic seven-cell hexagonal layout, as shown in Figure 2. User equipment is randomly and uniformly distributed within the cells, accounting for NLoS shadowing effects caused by obstacles. Key system parameters are listed in Table 1. In the comparison schemes, the random deployment strategy involves placing the 21 RISs randomly. For scenarios applying only the branch-bounding method and the proposed hybrid algorithm, the final deployment locations of the RISs are determined through algorithmic search.

**Figure 3:**
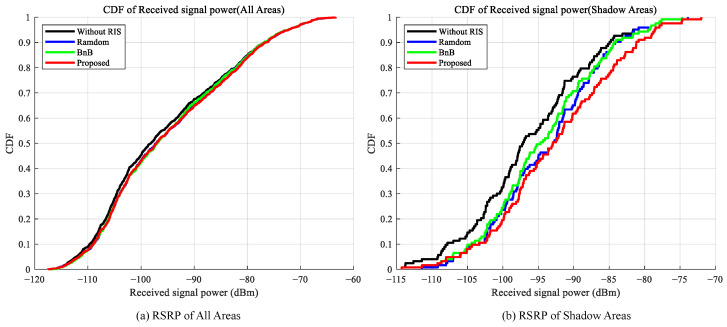
Comparison of RSRP Cumulative Distribution Functions (CDF). (**a**) Left figure shows all areas, (**b**) Right figure shows shaded areas.

First, we evaluate the impact of different deployment strategies on the reference signal receiving power (RSRP) for users. Figure 3 presents the CDF curves of RSRP for four scenarios: no RIS deployment (Baseline), random RIS deployment, RIS deployment locations determined solely by branch bounding, and RIS deployment locations determined using the proposed hybrid algorithm. The left figure displays the RSRP distribution across the entire area, while the right figure focuses on the shaded area. In the right figure, the curves for the three schemes in areas with poor signal quality show a significant rightward shift compared to the Baseline (no RIS deployment), indicating that RIS deployment substantially enhances user reception power. Within the shaded area, the proposed algorithm demonstrates a stronger advantage over the other schemes. The CDF curve for random deployment closely resembles that of the BnB deployment scheme. In contrast, the proposed hybrid optimization algorithm achieves more substantial gains in received signal power, particularly in the low-power range. This markedly improves the user experience in areas with poor signal coverage, demonstrating a powerful enhancement for users in weak coverage zones.

**Figure 4:**
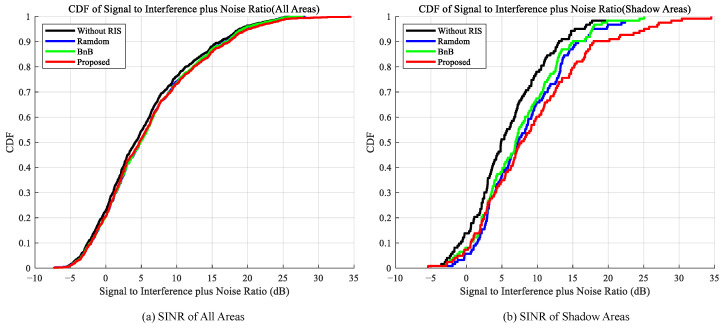
Comparison of SINR Cumulative Distribution Functions. (**a**) Left figure shows all areas, (**b**) Right figure shows shaded areas.

Furthermore, Figure 4 presents the CDF results of SINR under different deployment strategies. Similar to RSRP, the proposed algorithm achieves significant improvements in SINR performance. At the median point, the proposed algorithm elevates the SINR gain from a baseline of 4.88 dB to 7.72 dB, yielding an additional gain of 2.84 dB over the baseline scheme. In contrast, the additional gains achieved by the random deployment and BnB algorithms are only around 2.44 dB and 2.03 dB, respectively. This result demonstrates that the proposed algorithm not only enhances the signal but also effectively suppresses interference, thereby securing a significant advantage in improving actual communication quality.

**Figure 5:**
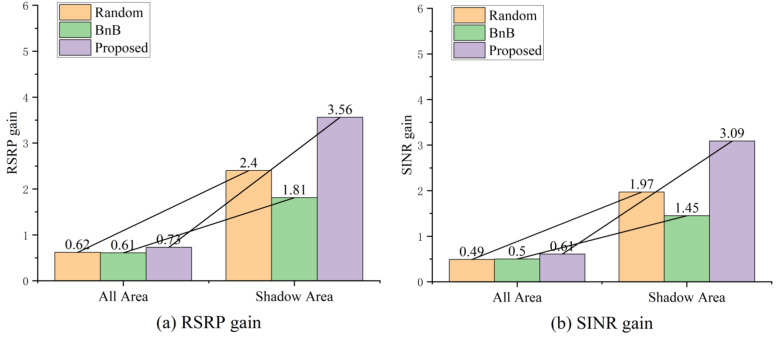
Comparison of RSRP and SINR Gains Across Different Schemes.

To more precisely quantify the performance of different deployment schemes in shadowed areas, Table 2 and Figure 5 present the comparison results of average RSRP and SINR. As shown in Table 2, the proposed algorithm demonstrates particularly significant performance advantages in shadowed areas, achieving an average SINR gain of 3.09 dB—approximately 56.85% higher than the 1.97 dB gain of the random deployment scheme. Simultaneously, the average RSRP gain also increases from 2.40 dB in random deployment to 3.56 dB, further validating the algorithm’s enhancement capability in weak coverage areas. As shown in Figure 5, the BnB algorithm consistently underperforms random deployment in both full-coverage and shadowed areas. This stems primarily from BnB’s tendency to concentrate RIS deployments in a small number of cells, resulting in uneven overall coverage distribution. For a 7-cell cellular system, this deployment bias significantly limits the average improvement in global performance, indirectly validating the importance and rationality of the coverage constraints designed in the proposed algorithm.

Similarly, Table 2 illustrates the impact of phase resolution on gain within the same deployment scheme. Lower resolutions introduce coarser phase quantisation granularity, leading to significant quantisation errors that constrain the RIS’s capability for precise alignment and signal enhancement. In our proposed method, the RSRP in the shadow areas is improved by 2.83 dB at 1-bit resolution compared to the baseline, while this gain increases to 3.56 dB at 2-bit resolution. The SINR gain also rises from 2.38 dB to 3.09 dB. This confirms that higher resolution indeed delivers superior system performance.

Compared to random deployment strategies and the BnB algorithm, the proposed hybrid algorithm demonstrates more stable performance gains. Although random deployment yields partial performance benefits in specific scenarios, its results are highly dependent on initial conditions, limiting overall optimisation effectiveness. The BnB algorithm alone fails to meet coverage fairness requirements within a reasonable range of time, resulting in global average performance improvements approaching those of random deployment. Conversely, the proposed hybrid algorithm achieves efficient resource allocation under coverage constraints through a two-stage mechanism: the first stage employs a greedy strategy to ensure global shadow area coverage fairness, while the second stage utilises branch-and-bound optimisation to further enhance overall system performance. Crucially, the first stage reduces the search space for the second stage, thereby lowering computational complexity. Experimental results demonstrate that this algorithm intelligently and efficiently utilises RIS resources, exhibiting significant advantages particularly in improving user experience in areas with weak coverage.

## 5. Conclusions and Future Work

This paper addresses the optimization problem of deploying reconfigurable intelligent surfaces under coverage constraints by proposing a hybrid optimization algorithm framework that integrates a greedy strategy with branch-and-bound methods to achieve this constrained deployment optimization. By decomposing the original NP-hard problem into two core subproblems, the proposed method balances computational efficiency and optimality in its algorithmic design: The first stage employs a greedy strategy to force-assign locally optimal RIS to each shadow area, strictly guaranteeing coverage completeness. The second stage then performs global optimization of the remaining RIS resources using the BnB algorithm to further enhance the overall system performance.

Simulation results demonstrate that the proposed algorithm delivers significantly superior performance improvements for users within shadowed areas compared to random deployment strategies and the BnB algorithm. For users who are in shadowed zones, this method achieves an average signal-to-interference-plus-noise ratio (SINR) gain of 3.09 dB. This represents an improvement of nearly 56.85% over the 1.97 dB gain achieved by random deployment, fully validating its effectiveness in addressing coverage fairness and enhancing system performance. Moreover, this algorithm transforms the original problem’s exponentially unsolvable complexity into an engineering-solvable polynomial-level complexity. These results further demonstrate the critical synergy of the two-stage design: the greedy stage ensures baseline quality of service, while the branch-bounding stage achieves global performance optimization under constraints. Future work will focus on extending this algorithm to dynamically evolving network environments and exploring the joint optimization of deployment locations and beamforming.

## Figures and Tables

**Table 1:** Simulation parameters.

Parameter	Value
Layout	Seven-cell hexagonal
Station spacing	500 m
UE distribution	Uniform random
NLoS shadow areas	1–3 per cell (random)
Carrier frequency	2.6 GHz
Bandwidth	100 MHz
Path loss	3GPP UMa
BS transmit power	46 dBm
BS antenna height	25 m
RIS count	21
RIS size	40×40 elements
RIS height	15 m
RIS phase quantisation bits	2 bits

**Table 2:** Performance comparison of different RIS deployment methods (dB).

Phase Resolution	Index	Area	Baseline	Random		BnB		Proposed	
			Without RIS	With RIS	Gain	With RIS	Gain	With RIS	Gain
1 bit	RSRP	All Area	−95.39	−94.96	0.42	−94.95	0.44	−94.85	0.53
		Shadow Area	−96.11	−94.44	1.67	−94.80	1.32	−93.28	2.83
	SINR	All Area	5.60	5.93	0.33	5.97	0.37	6.03	0.42
		Shadow Area	6.01	7.31	1.30	7.06	1.04	8.40	2.38
2 bits	RSRP	All Area	−95.39	−94.77	0.62	−94.77	0.61	−94.65	0.73
		Shadow Area	−96.11	−93.72	2.40	−94.31	1.81	−92.55	3.56
	SINR	All Area	5.60	6.09	0.49	6.11	0.50	6.21	0.61
		Shadow Area	6.01	7.99	1.97	7.46	1.45	9.10	3.09

## Data Availability

The original contributions presented in this study are included in the article. Further inquiries can be directed to the corresponding author.
